# Identification of SLC41A3 as a novel player in magnesium homeostasis

**DOI:** 10.1038/srep28565

**Published:** 2016-06-28

**Authors:** Jeroen H.F. de Baaij, Francisco J. Arjona, Michiel van den Brand, Marla Lavrijsen, Anke L.L. Lameris, René J.M. Bindels, Joost G.J. Hoenderop

**Affiliations:** 1 Department of Physiology Radboud Institute for Molecular Life Sciences, Radboud university medical center, Nijmegen, The Netherlands; 2 Department of Pathology, Radboud Institute for Molecular Life Sciences, Radboud university medical center, Nijmegen, The Netherlands

## Abstract

Regulation of the body Mg^2+^ balance takes place in the distal convoluted tubule (DCT), where transcellular reabsorption determines the final urinary Mg^2+^ excretion. The basolateral Mg^2+^ extrusion mechanism in the DCT is still unknown, but recent findings suggest that SLC41 proteins contribute to Mg^2+^ extrusion. The aim of this study was, therefore, to characterize the functional role of SLC41A3 in Mg^2+^ homeostasis using the *Slc41a3* knockout (*Slc41a3*^−/−^) mouse. By quantitative PCR analysis it was shown that *Slc41a3* is the only SLC41 isoform with enriched expression in the DCT. Interestingly, serum and urine electrolyte determinations demonstrated that *Slc41a3*^−/−^ mice suffer from hypomagnesemia. The intestinal Mg^2+^ absorption capacity was measured using the stable ^25^Mg^2+^ isotope in mice fed a low Mg^2+^ diet. ^25^Mg^2+^ uptake was similar in wildtype (*Slc41a3*^+/+^) and *Slc41a3*^−/−^ mice, although *Slc41a3*^−/−^ animals exhibited increased intestinal mRNA expression of Mg^2+^ transporters *Trpm6* and *Slc41a1*. Remarkably, some of the *Slc41a3*^−/−^ mice developed severe unilateral hydronephrosis. In conclusion, SLC41A3 was established as a new factor for Mg^2+^ handling.

The distal convoluted tubule (DCT) is essential for magnesium (Mg^2+^) homeostasis^1^. Although only 10% of the filtered Mg^2+^ is reabsorbed in this segment of the kidney, it determines the final urinary Mg^2+^ excretion since no reabsorption takes place beyond this segment^1^. In the DCT, Mg^2+^ enters the cell via the Transient Receptor Potential Melastatin type 6 (TRPM6) divalent cation channel^2^, while the basolateral Mg^2+^ extrusion mechanism remains to be identified. Disturbances in this transcellular transport process result in hypomagnesemia, which is characterized by a variety of clinical symptoms including cardiac arrhythmias, muscle cramps and fatigue^1^. Additionally, drugs directly affecting the DCT such as epidermal growth factor receptor inhibitors, calcineurin inhibitors, and diuretics reduce blood Mg^2+^ levels^3^. Therefore, understanding the molecular mechanism underlying Mg^2+^ reabsorption in the DCT is of major importance for the treatment of patients with hypomagnesemia.

In a recent transcriptome screening study, *Solute carrier family 41 member 3* (*Slc41a3*) was identified as a highly regulated gene by dietary Mg^2+^ intake^4^. In this study mice were fed with Mg^2+^-enriched or Mg^2+^-deficient diets after which the DCT segments were isolated. Using a quantitative gene expression microarray, the Mg^2+^-sensitive genes in the DCT were identified. *Slc41a3* was among the most significant genes with increased expression in response to a Mg^2+^-deficient diet.

SLC41A3 was originally described by Quamme and colleagues as part of the solute carrier family 41 of putative Mg^2+^ transporters^5^. Although SLC41A3 has never been studied in detail, a few reports have addressed the structure, regulation and function of its close homologue SLC41A1 (52% identity at amino acid level)^6^,^7^,^8^. There is controversy about the structure of SLC41A1; some groups support a 10 transmembrane domain structure, while others suggest 11 transmembrane domains with an extracellular carboxyl (C)-terminus^8^,^9^. At the functional level, initial experiments in *Xenopus laevis* oocytes demonstrated Mg^2+^ currents for SLC41A1 and SLC41A3 using physiological Mg^2+^ concentrations, suggesting a channel-like function^5^,^10^. Indeed, SLC41 proteins posses conserved pore regions with the MgtE bacterial Mg^2+^ channel^8^,^11^,^12^. In contrast, recent experiments using Mg^2+^-sensitive fluorescent probes showed Na^+^-dependent Mg^2+^ transport, suggesting that SLC41A1 may be the long-sought Na^+^/Mg^2+^-exchanger^7^,^13^. Interestingly, an exon-skipping mutation in *SLC41A1* resulted in a nephronophthisis-like phenotype in a patient eventually requiring renal transplantation^14^. Whether SLC41A3 may have a similar function as SLC41A1 has never been examined. Interestingly, in contrast to *Slc41a3*, Mg^2+^-sensitive regulation of *Slc41a1* expression was not observed in the DCT transcriptome study^4^.

The aim of the present study was, therefore, to characterize the role of SLC41A3 in renal and intestinal Mg^2+^ (re)absorption. For this purpose, *Slc41a3* knockout mice (*Slc41a3*^−/−^) were analysed for electrolyte homeostasis, intestinal function and renal abnormalities. Furthermore, by challenging the mice with Mg^2+^-deficient diets, compensatory mechanisms in intestine, kidney and brain were examined in detail. Intestinal Mg^2+^ absorption studies were performed using the ^25^Mg^2+^ isotope to address the functional role of SLC41A3 in the intestine.

## Results

### Expression profile of Slc41 isoforms in kidney and DCT

**Figure 1:**
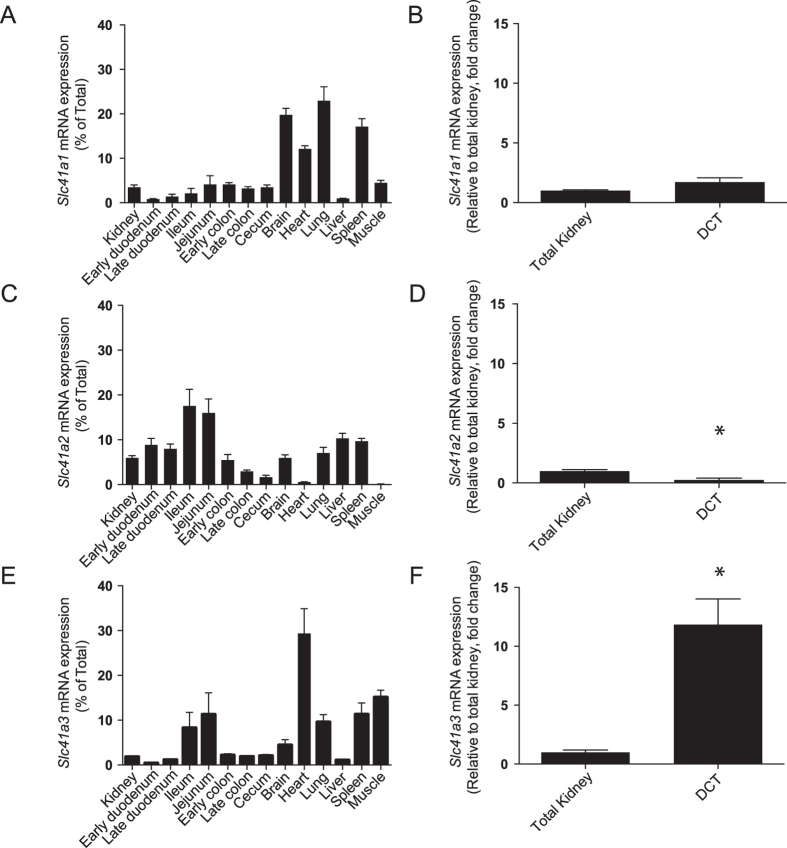
*Slc41a3* expression is enriched in the distal convoluted tubule (DCT). (**A–F**) mRNA expression levels of *Slc41a1* (**A,B**), *Slc41a2* (**C,D**) and *Slc41a3* (**E,F**) in a panel of mouse tissues (**A,C,E**) and in COPAS-sorted DCT cells (**B,D,F**) were measured by RT-qPCR. Relative gene expression was analyzed using the Livak method (2^−ΔΔCt^), where results are normalized against *Gapdh* expression (reference gene). Data represent the mean of three individual experiments ± SEM and are expressed as the percentage of the total tissue gene expression (**A,C,E**) or as the fold change relative to total kidney gene expression (**B,D,F**). ^*^P < 0.05 indicates a significant difference from total kidney gene expression.

To examine the role of the SLC41 protein family in renal Mg^2+^ transport, a tissue expression screening for *Slc41a1*, *Slc41a2* and *Slc41a3* was performed using RT-quantitative PCR (RT-qPCR, Fig. 1). All three isoforms showed a ubiquitous expression pattern. Robust *Slc41a1* expression was detected in brain, heart and lung, *Slc41a2* was predominantly expressed in the proximal intestine whereas *Slc41a3* expression was highest in heart, lung and small intestine. However, in RT-PCR analysis of isolated DCT segments *Slc41a3* messenger RNA (mRNA) transcript levels showed a significant 10-fold enrichment compared to total kidney mRNA. *Slc41a1* and *Slc41a2* expression was not enriched in DCT compared to other segments, while the latter transporter was even significantly decreased, suggesting that SLC41A3 is the most relevant SLC41 family member for Mg^2+^ reabsorption in the DCT.

### Slc41a3 mouse breeding

**Figure 2:**
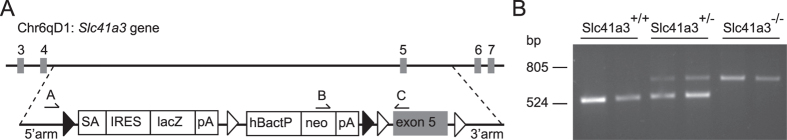
Characteristics of the *Slc41a3*^−/−^ mouse. (**A**) Targeted insertion of the knockout cassette. Top: *Slc41a3* locus on chromosome 6. Bottom: targeted allele in which the knockout cassette is inserted before exon 5. Grey boxes indicate exons; black triangles depict LoxP sites; white triangles depict Flp sites; arrows depict genotype primers (A–C). SA: splice acceptor, IRES: interinal ribosome entry site, lacZ: ß-galactosidase, pA: polyA, hBactP: promoter, neo: neomycin resistance gene. (**B**) Identification of the mouse genotype by PCR analysis of ear-derived DNA. The PCR product in the upper gel shows the presence of the wild-type allele (+/+) using primers A and C; the lower gel shows the knockout allele (–/–) using primers (B,C). Both alleles are detected in heterozygous animals (+/–).

To examine the function of SLC41A3 in Mg^2+^ homeostasis, *Slc41a3*^−/−^ mice were generated. Breeding of heterozygous (*Slc41a3*^+/−^) mice resulted in a normal Mendelian inheritance pattern in the offspring. Of a total of 181 mice, 32% were genotyped *Slc41a3*^+/+^, 46% *Slc41a3*^+/−^ and 22% *Slc41a3*^−/−^. Since Shirpa (**S**mithKline Beecham, **H**arwell, **I**mperial College, **R**oyal London Hospital, **p**henotype **a**ssessment) screening by the Mouse Genetics Project reported abnormal locomotor coordination of *Slc41a3*^−/−^ mice fed with a high fat diet (MGI: 1918949)^15^, special attention to behavioral observations was given during the breeding procedure. However, no abnormalities in behavior of the mice including ataxia and locomotor behavior were observed. By visual inspection, it was not possible to distinguish between *Slc41a3*^+/+^, *Slc41a3*^+/−^ and *Slc41a3*^−/−^ mice based on behavior or external phenotype. All offspring was genotyped for the insertion of the knockout cassette and presence of the wild type *Slc41a3* allele (Fig. 2B).

### Hypomagnesemia in *Slc41a3* ^−/−^ mice

**Figure 3:**
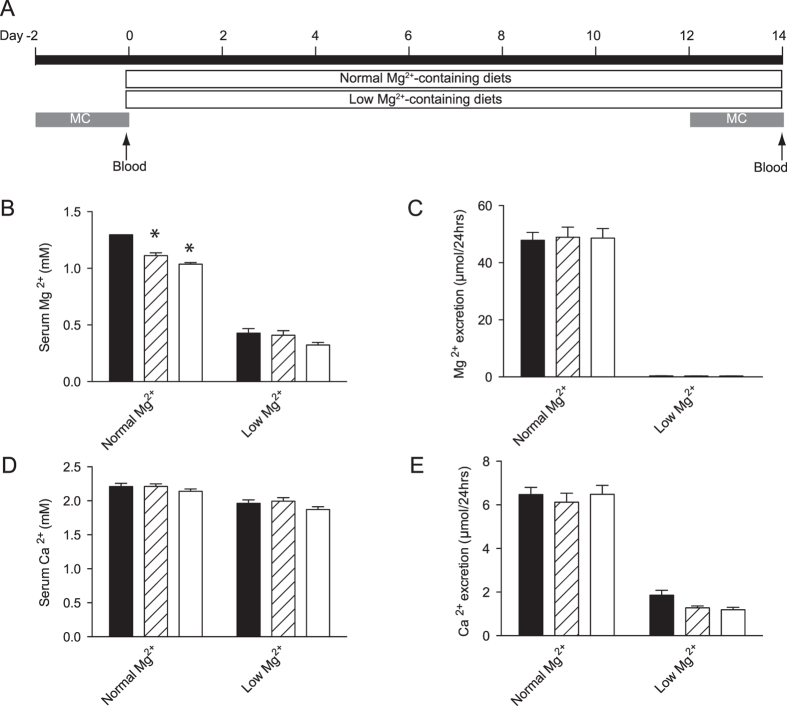
*Slc41a3*^−/−^ mice suffer from isolated hypomagnesemia. (**A**) *Slc41a3*^+/+^, *Slc41a3*^+/−^, *Slc41a3*^−/−^ mice were randomly assigned to the low or normal Mg^2+^ group, each group consisting of 10 animals. Before the start of the experiment, the mice were housed in metabolic cages for urine and feces collection for 48 hours and blood was taken. Subsequently, mice were fed with the Mg^2+^ diets for 14 days of which animals were housed in metabolic cages during the last 48 hours for urine and feces sampling. At the end of the experiments, the animals were sacrificed and blood and organs were taken for further analysis. B-E, Serum Mg^2+^ (**B**) and Ca^2+^ (**D**) concentrations of *Slc41a3*^+/+^ (black bars), *Slc41a3*^+/−^ (grey bars) and *Slc41a3*^−/−^ (white bars) mice fed with a low or normal Mg^2+^-containing diet for 14 days. 24 hour urinary Mg^2+^ (**C**) and Ca^2+^ (**E**) excretion at day 14 of *Slc41a3*^+/+^ (black bars), *Slc41a3*^+/−^ (grey bars) and *Slc41a3*^−/−^ (white bars) mice fed with a low or normal Mg^2+^-containing diet for 14 days. Values are presented as means ± SEM (n = 10). *P < 0.05 is considered statistically significant compared to *Slc41a3*^+/+^ mice fed the same diet.

To investigate the role of SLC41A3 in Mg^2+^ homeostasis, mice were subjected to normal or low Mg^2+^-containing diets for 14 days. Blood, 24-hour urine and feces were collected at day 0 and 14 of the experiment using metabolic cages (Fig. 3A). At the end of the experiment, the mice were sacrificed and tissues were collected for further analysis. No significant differences were observed between the body weights of *Slc41a3*^+/+^ mice and *Slc41a3*^+/−^ and *Slc41a3*^−/−^ littermates (Table 1). Food and water intake, urinary volume and fecal excretion were comparable among all mice genotypes on the same diet (Table 1). On the normal diet, serum Mg^2+^ concentrations of *Slc41a3*^−/−^ mice were significantly decreased by 29 ± 2% compared to *Slc41a3*^+/+^ mice (1.30 ± 0.05 mM vs. 1.04 ± 0.01 mM, Fig. 3B). Urinary Mg^2+^ excretion was not different (Fig. 3C). Furthermore, both serum and urinary calcium (Ca^2+^) levels were not significantly altered in *Slc41a3*^−/−^ mice compared to *Slc41a3*^+/+^ mice, underlining the specificity of the hypomagnesemia in these *Slc41a3*^−/−^ mice (Fig. 3D,E). *Slc41a3*^+/+^ mice fed a Mg^2+^-deficient diet for two weeks, displayed hypomagnesemia (serum Mg^2+^ levels of 0.43 ± 0.04 mM, Fig. 3B). Although the serum Mg^2+^ levels of *Slc41a3*^−/−^ mice on a low Mg^2+^ diet were lower (0.32 ± 0.02 mM) and decreased to a similar extent as the *Slc41a3*^−/−^ mice on the normal Mg^2+^ diet (26%), they did not reach a significant difference compared to *Slc41a3*^+/+^ mice (p = 0.14). On the low Mg^2+^ diet, no differences in urinary Mg^2+^ and Ca^2+^ excretion or serum Ca^2+^ concentrations were measured among the groups (Fig. 3C–E).

### Hydronephrosis in subset of *Slc41a3* ^−/−^ mice

**Figure 4:**
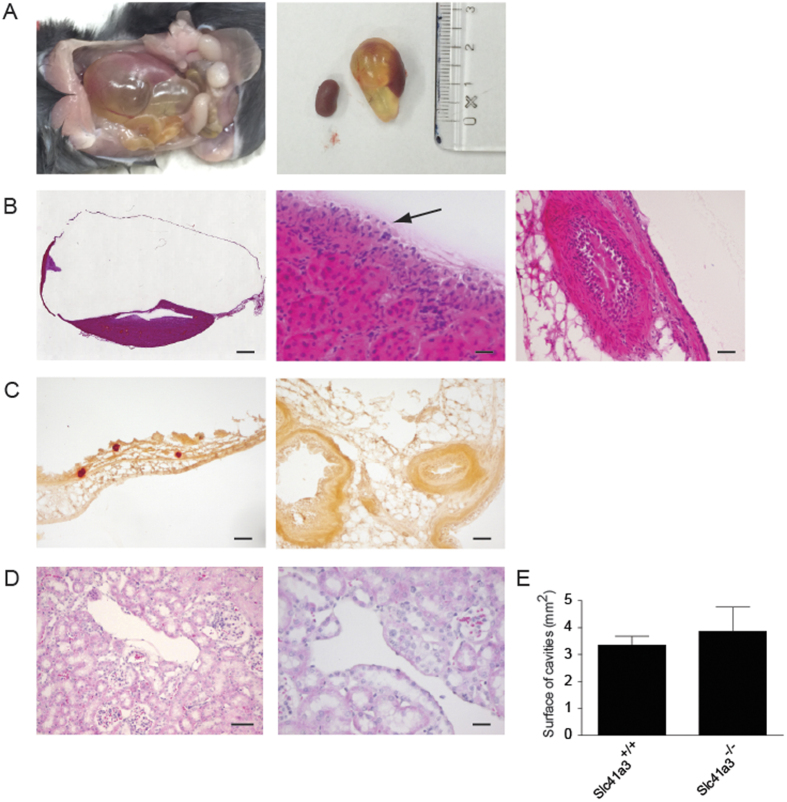
*Slc41a3*^−/−^ mice develop hydronephrosis on low Mg^2+^ diets. (**A**) Images of hydronephrotic kidneys found in 2 out of 20 *Slc41a3*^−/−^ mice fed low Mg^2+^ diets. (**B**) H&E stainings of the hydronephrosis kidney detected in *Slc41a3*^−/−^ mice showing transitional epithelium lining the hydronephrosis. The left image gives an overview of the total kidney (bar: 1 mm). The middle image shows the transitional epithelium with a black arrow indicating the umbrella cells (bar: 20 μm). The right image shows a slender proximal ureter filled with eosinophilic material, indicating stasis (bar: 20 μm). (**C**) Alizirin red staining of the hydronephrosis kidney did not show Ca^2+^ deposits (bar: μm). (**D**) H&E stainings of normal kidney tissue from *Slc41a3*^−/−^ mice fed with normal Mg^2+^ demonstrating venous and tubular dilations (left image bar: 100 μm, right image bar: 20 μm). (**E**) Quantification of dilated surface in *Slc41a3*^+/+^ and *Slc41a3*^−/−^ mice. Values are presented as means ± SEM (n = 10).

Interestingly, in 10% of the male *Slc41a3*^−/−^ mice on the low Mg^2+^ diet a severely increased kidney size was observed in the left kidney caused by a hydronephrosis (Fig. 4A). The kidney volume was 6–8x larger than the other kidney of the same animal and caused a serious organ rearrangement in the peritoneal cavity. The unilateral hydronephrotic kidney was observed in 10% of the *Slc41a3*^−/−^ mice in the low Mg^2+^ group and was never detected in *Slc41a3*^−/−^ mice fed the normal Mg^2+^ diet or in the *Slc41a3*^+/+^ mice. To further assess the morphology of the hydronephrosis, the kidney tissue was stained using standard hematoxylin and eosin (H&E) stainings (Fig. 4B, left image). The presence of transitional epithelium around the dilated tissue and the absence of dilated ureters suggested that the origin of the hydronephrosis is located in the renal calyx or renal pelvis. Indeed, umbrella cells were detected to further confirm the urothelial origin of the tissue (Fig. 4B, middle image). However, major parts of the tissue lining the fluid-filled cavity were not covered with transitional epithelium, but existed of fibrous connective tissue with flattened epithelium or absence of epithelium. The ureter immediately next to the cyst was slender and filled with eosinophilic material, suggesting an obstruction at the pyelo-ureteral junction and absence of urinary flow (Fig. 4B, right image). However, no clear anatomical cause for obstruction could be identified. To access whether more initial stages of the development of the hydronephrosis could be detected, kidneys from *Slc41a3*^−/−^ mice fed with the normal Mg^2+^ diet without apparent abnormalities at the exterior of the kidney were processed using a standard H&E staining (Fig. 4C). Although a subset of tubules and blood vessels displayed some dilatation in the cortex and the medulla of *Slc41a3*^+/+^ and *Slc41a3*^−/−^ mice, quantification of the surface size of these dilations did not show significant differences between kidneys from *Slc41a3*^+/+^ and *Slc41a3*^−/−^ mice (Fig. 4D).

### Expression of Mg^2+^ transporters in kidney and colon of Slc41a3^−/−^ mice

**Figure 5:**
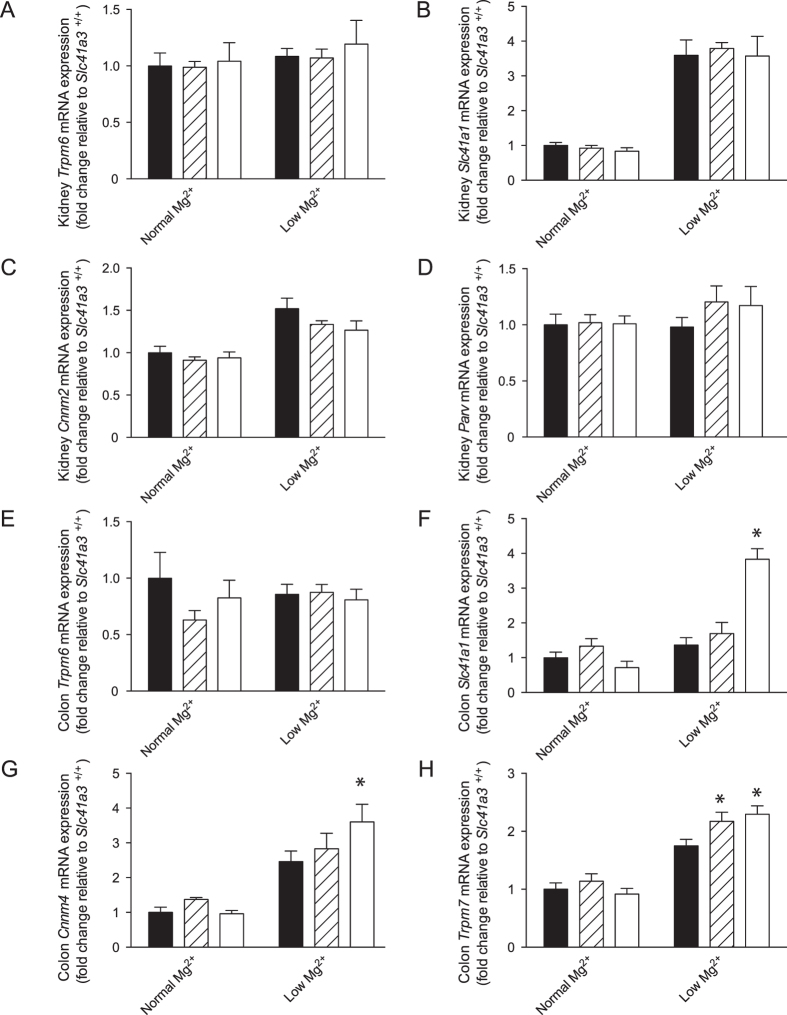
Compensatory mechanisms for the loss of Slc41a3. (**A–H**) The mRNA expression levels of *Trpm6* (**A,E**), *Slc41a1* **(B,F**), *Cnnm2* (**C**), *Parvalbumin* (**D**), *Cnnm4* (**G**) and *Trpm7* (**H**) in kidney (**A–D**) or colon (**E–H**) of *Slc41a3*^+/+^ (black bars), *Slc41a3*^+/−^ (striped bars) and *Slc41a3*^−/−^ (white bars) mice fed with a low or a normal Mg^2+^-containing diet for 14 days were measured by RT-qPCR. Relative gene expression was analyzed using the Livak method (2^−ΔΔCt^), where results are normalized against *Gapdh* expression (reference gene). Data represent means ± SEM (n = 10) and are expressed as fold difference when compared to the gene expression in normal diet fed *Slc41a3*^+/+^ mice. ^*^P < 0.05 indicates a statistically significance compared to *Slc41a3*^+/+^ mice fed the same diet.

The kidney and colon are the main sites of Mg^2+^ (re)absorption. To examine the compensatory mechanisms of the renal Mg^2+^ wasting in *Slc41a3*^−/−^ mice, the expression level of renal Mg^2+^ transporters was analyzed using RT-qPCR (Fig. 5). The mRNA transcript levels of *Trpm6*, *Slc41a1*, *Cnnm2* and *Parvalbumin* were not significantly different between *Slc41a3*^+/+^ and *Slc41a3*^−/−^ mice (Fig. 5A–D). Renal *Slc41a1* expression was increased in the low Mg^2+^ diet group mice compared to mice on the normal Mg^2+^ diet for all genotypes (Fig. 5B). Furthermore, the renal mRNA levels of *Egf, Ncc, Cldn16 and Cldn19* were not altered in *Slc41a3*^−/−^ mice in comparison with their *Slc41a3*^+/+^ littermates (Supplementary Fig. 1). Subsequently, the expression of Mg^2+^ transporters in the colon, where Mg^2+^ is actively absorbed from the diet, was examined by RT-qPCR. Particularly, in the low Mg^2+^ groups, gene expression levels of *Slc41a1*, *Cnnm4* and *Trpm7* were increased in the colon of *Slc41a3*^−/−^ animals compared to colon of their *Slc41a3*^+/+^ littermates (Fig. 5E–H). In contrast, no significant difference in colonic *Trpm6* expression was detected between *Slc41a3*^+/+^ and *Slc41a3*^−/−^ mice. Since *Slc41a3* is also expressed in brain (Fig. 1) and *SLC41A1* is associated with Parkinson’s disease^16^,^17^, the expression levels of *Trpm7* and *Slc41a1* in the brain were determined by RT-qPCR. No significant changes in brain mRNA expression levels of *Trpm7* and *Slc41a1* were observed among the genotypes (Supplementary Fig. 2).

### Intestinal Mg^2+^ absorption is similar in Slc41a3^+/+^ and Slc41a3^−/−^ mice

**Figure 6:**
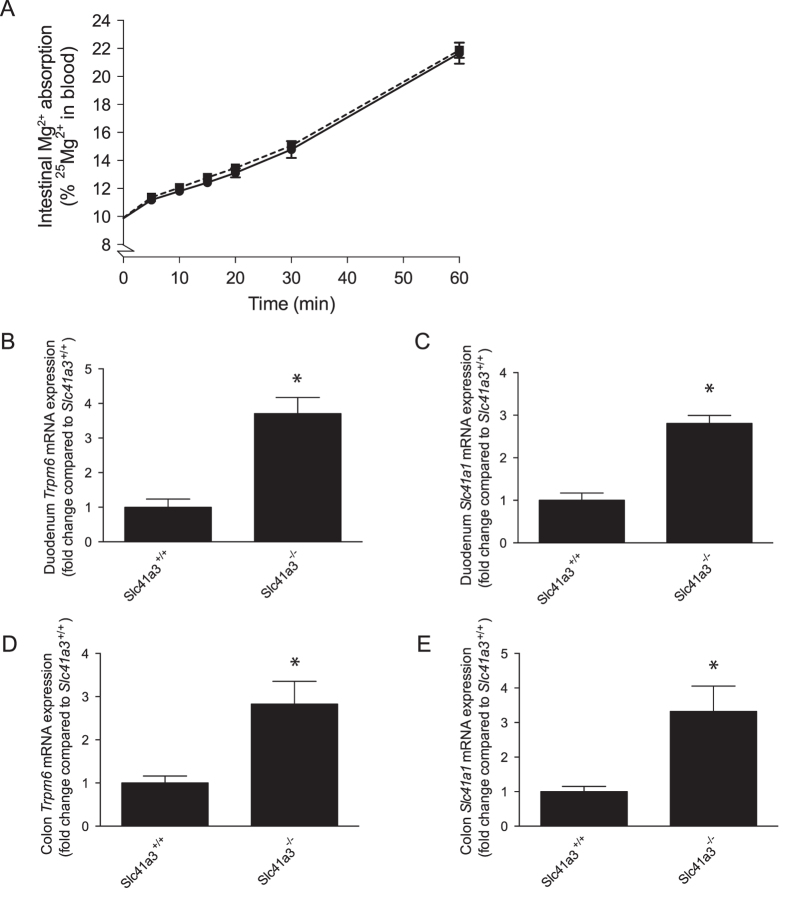
Increased expression of intestinal Mg^2+^ transporters compensate for Slc41a3 KO. (**A**) 60 minutes intestinal ^25^Mg^2+^ absorption in *Slc41a3*^+/+^ (solid line) and *Slc41a3*^−/−^ (dashed line) mice after 10 days on low Mg^2+^ diets. B-E, The mRNA expression levels of *Trpm6* (**B,D**) and *Slc41a1* (**C,E**) in duodenum (**B,C**) or colon (**D,E**) of *Slc41a3*^+/+^ and *Slc41a3*^−/−^ mice fed with a low Mg^2+^-containing diet for 10 days were measured by RT-qPCR. Relative gene expression was analyzed using the Livak method (2^−ΔΔCt^), where results are normalized against *Gapdh* expression (reference gene). Values are presented as means ± SEM (n = 10) and are expressed as fold difference when compared to the gene expression in normal diet fed *Slc41a3*^+/+^ mice. ^*^P < 0.05 is considered statistically significant compared to *Slc41a3*^+/+^ mice.

To further examine the role *Slc41a3*^−/−^ in the intestine, the Mg^2+^ absorption capacity was determined using the stable ^25^Mg^2+^ isotope^18^. The mice were subjected to 10 days of low Mg^2+^ diet prior to the ^25^Mg^2+^ absorption analysis. At the final day of the experiment, the mice were administrated ^25^Mg^2+^ by oral gavage and subsequently blood was taken from the tail up to 60 minutes after the administration. The natural abundance of ^25^Mg^2+^ in blood is 10%, which was doubled during 60 minutes of ^25^Mg^2+^ uptake to more than 20% (Fig. 6A). However, no significant differences in Mg^2+^ absorption were detected between *Slc41a3*^+/+^ and *Slc41a3*^−/−^ mice (Fig. 6A). Determination of the mRNA expression levels of *Trpm6* and *Slc41a1* in intestinal Mg^2+^ uptake showed that the expression of both genes is magnified in duodenum and colon of *Slc41a3*^−/−^ mice (Fig. 6B–E).

## Discussion

This study identified SLC41A3 as a novel player in Mg^2+^ homeostasis. This conclusion is based on the following results: *i) Slc41a3* is specifically expressed in the DCT and in the intestine where Mg^2+^ is (re)absorbed; *ii) Slc41a3*^−/−^ mice suffer from hypomagnesemia and normomagnesiuria indicating a possible renal Mg^2+^ leak; *iii)* intestinal Mg^2+^ transporters including *Trpm6* and *Slc41a1* are upregulated in *Slc41a3*^−/−^ mice. Additionally, this study shows that some *Slc41a3*^−/−^ mice fed a Mg^2+^-deficient diet develop hydronephrosis.

The urinary electrolyte levels in the hypomagnesemic *Slc41a3*^−/−^ mice point to a specific renal Mg^2+^ leak, mimicking the phenotype of patients with renal Mg^2+^ wasting^19^,^20^,^21^, namely low serum Mg^2+^ levels accompanied by normal urinary Mg^2+^ excretion. Under normal physiological circumstances, the kidney should be able to compensate for reduced blood Mg^2+^ levels by increasing renal Mg^2+^ reabsorption. However, the *Slc41a3*^−/−^ mice failed to counteract their urinary Mg^2+^ wasting. Moreover, the urinary Mg^2+^ excretion was reduced in all mice fed with Mg^2+^-deficient diets, showing that *Slc41a3*^−/−^ mice still have the ability to raise renal Mg^2+^ reabsorption despite inactivation of SLC41A3. However, in *Slc41a3*^−/−^ fed a low Mg^2+^ diet for 14 days, serum Mg^2+^ concentrations were 26% lower than in *Slc41a3*^+/+^ littermates. Although this finding did not reach statistically significance (p = 0.14), this suggests that the Mg^2+^ reabsorption capacity is impaired in *Slc41a3*^−/−^ mice fed the Mg^2+^-deficient diet. However, longer treatment may be necessary to observe a significant reduction of serum Mg^2+^ concentrations. Additionally, the intestinal ^25^Mg^2+^ absorption was normal in *Slc41a3*^−/−^ mice, potentially because increased *Trpm7* and *Slc41a1* expression compensates for the loss of SLC41A3 function.

In kidney, *Slc41a3* was, in contrast to its close homologue *Slc41a1*, highly enriched in DCT. Likewise, the expression of *Slc41a3*, but not of *Slc41a1*, in DCT is highly dependent on dietary Mg^2+^ intake^4^. These results are comparable to the established findings concerning the Mg^2+^ channels TRPM6 and TRPM7. TRPM6 provides the specific luminal Mg^2+^ uptake mechanism in kidney and intestine^2^,^22^. Hence, the expression of TRPM6 is localized to the DCT and the colon where it is highly regulated by dietary Mg^2+^ availability, EGF, insulin, ATP and estrogens^22^,^23^,^24^,^25^,^26^. In contrast, TRPM7 is ubiquitously expressed and is involved in basic cellular Mg^2+^ homeostasis^27^,^28^. Its expression is insensitive to dietary Mg^2+^ changes^22^. A comparable concept could apply to SLC41 proteins, in which SLC41A3 would be specific for epithelial Mg^2+^ uptake in the DCT and SLC41A1 would serve as ubiquitously expressed general Mg^2+^ transporter.

The molecular function of SLC41A3 in the DCT remains elusive. SLC41A3 activity has only been examined in *Xenopus laevis* oocytes by voltage-clamp, showing Mg^2+^ currents with a K_m_ within the physiological range for blood Mg^2+^ concentrations^5^. However, when overexpressed in human embryonic kidney 293 (HEK293) cells Mg^2+^ currents could not be detected (unpublished data from our lab). The function of the close homologue SLC41A1 has been examined in more detail. As SLC41A3, SLC41A1 mediates Mg^2+^ currents in *Xenopus laevis* oocytes^10^. Moreover, its Mg^2+^ transport capacity was further established in the TRPM7-deficient DT40 cell line, where SLC41A1 restored cell growth^29^. Conversely, SLC41A3 failed to complement TRPM7-deficient DT40 cells, suggesting that SLC41A3 transport activity may depend on partner proteins. Importantly, recent data suggest that SLC41A1 acts as a Na^+^/Mg^2+^-exchanger being localized at the basolateral plasma membrane^7^,^14^. Although SLC41A1 and SLC41A3 are very homologous and may have a similar mode of action, definitive evidence for the function and plasma membrane localization of SLC41A3 is currently lacking. Future cellular and functional studies should confirm the Mg^2+^ transporting function of SLC41A3.

Although *Slc41a3* is expressed in the intestine, *Slc41a3*^−/−^ mice exhibited normal intestinal Mg^2+^ absorption compared to their *Slc41a3*^+/+^ littermates. However, the expression of Mg^2+^ transporters *Slc41a1* and *Trpm6* was increased in duodenum of *Slc41a3*^−/−^ mice, suggesting that loss of SLC41A3 function induces a compensatory mechanism to facilitate normal Mg^2+^ absorption. Specifically, the upregulation of *Slc41a1* is of interest because the structure of SLC41A1 is largely similar to SLC41A3^8^. Importantly, the pore region of SLC41A1 and SLC41A3 proteins is conserved from MgtE bacterial Mg^2+^ channels and essential for their function^8^,^11^,^12^. If the increased expression of *Slc41a1* indeed compensates for defects in intestinal Mg^2+^ absorption in *Slc41a3*^−/−^ mice, this would suggest that SLC41A1 and SLC41A3 are functionally redundant. Future studies with *Slc41a1* and *Slc41a3* double knockout mice should substantiate the hypothesis that SLC41A1 and SLC41A3 function cooperatively to regulate Mg^2+^ handling.

The development of hydronephrosis in *Slc41a3*^−/−^ mice is a striking finding of our study. H&E stainings showed transitional epithelia including umbrella cells lining the hydronephrotic region, excluding a cystic origin of the kidney malformation^30^. However, important parts of the lining tissue of the hydronephrosis were not covered with transitional epithelium, but with a layer of fibrous connective tissue that normally originates from the renal capsule. A similar phenotype of perinephric pseudocysts, in which fluid accumulates in a fibrous sac surrounding the kidney, has been observed previously in humans, mice and cats^31^,^32^,^33^. In a previously described mouse study using the C57BL/6J strain, extensive histological analysis showed that unilateral perinephric pseudocyst can be formed from an initial hydronephrosis that at some point ruptures when the integrity of the lining wall is compromised^31^. The urine may then leak into the subcapsular space between the renal capsule and the remnant kidney. In concordance with these findings, histological analysis of the *Slc41a3*^−/−^ mice demonstrated both transitional epithelium and connective tissue lining the cavity, suggesting that an initial hydronephrosis may have ruptured resulting in a perinephric pseudocyst.

Hydronephrosis is normally the consequence of obstruction of the ureter resulting in fluid retention in the renal calyx and pelvis^34^. Anatomical obstructions were not found in *Slc41a3*^−/−^ mice and, therefore, the cause of the hydronephrosis could not be identified. Importantly, only a subset of the *Slc41a3*^−/−^ mice developed hydronephrosis, suggesting that *Slc41a3*^−/−^ mice are more sensitive to the development of hydronephrosis, but that inactivation of Slc41a3 is not causative of hydronephrosis *per se*. Speculatively, the dietary Mg^2+^ availability could have contributed to the development of the hydronephrosis. It is widely acknowledged that Mg^2+^ can prevent urolithiasis by reducing the formation of calcium oxalate (CaC_2_O_4_) and calcium phosphate (Ca(H_2_PO_4_)_2_) stones and deposits^35^,^36^,^37^. Urolithiasis can cause obstruction of the ureter and, therefore, result in hydronephrosis^38^. However, alirizin red stainings did not show Ca^2+^ deposits in *Slc41a3*^−/−^ mice on the low Mg^2+^ diet despite their low urinary Mg^2+^ excretion. H&E stainings demonstrated a slender proximal ureter containing amorphic eosinophilic material, possibly due to stasis. Although the slender proximal ureter suggests a stenosis at the ureteropelvic junction, no anatomical cause for obstruction could be identified. It could be hypothesized that a functional defect in peristalsis caused hydronephrosis in *Slc41a3*^−/−^ mice, but to substantiate this further studies are required.

Interestingly, a mutation of *SLC41A1* was recently shown to be causative for a nephronophthisis-like (NPHP-like) phenotype in an Italian family^14^. The kidneys of the patients showed irregular echogenicity when examined by kidney ultrasonography^14^, suggesting renal cysts or hydronephrosis. However, hydronephrosis could be excluded by subsequent histological analysis, which showed periglomerular fibrosis, tubular ectasia, tubular basement membrane disruption and tubulointerstitial infiltrations^14^. The aforementioned signs of inflammation and fibrosis were absent in the remnant kidney tissue of *Slc41a3*^−/−^ mice. Moreover, in contrast to the NPHP-like phenotype in patients with SLC41A1 mutations, the kidney size was markedly increased in the *Slc41a3*^−/−^ mice that suffered from hydronephrosis. More patients with mutations in SLC41A1 should be identified and examined for hydronephrosis to allow final conclusions on the kidney phenotype of these patients.

In conclusion, our study of the *Slc41a3* mice has identified SLC41A3 as a novel player in Mg^2+^ (re)absorption and potentially a new factor in the formation of hydronephrosis. Consequently, *SLC41A3* should be included in genetic screenings for hypomagnesemic patients.

## Methods

### Expression analysis

Three C57BL/6 mice were sacrificed; kidney, duodenum, ileum, jejunum, cecum, colon, brain, lung, liver, spleen, muscle, and heart tissues were collected. For the collection of DCT material, transgenic parvalbumin-eGFP mice were used as described previously (kind gift from Dr. Monyer, University of Heidelberg, Germany)^39^. In short, mice were anesthetized by a mixture injection of domitor (0.01 mg/g of body weight) and ketamine (0.1 mg/g of body weight). Subsequently, the mice were perfused with 10 ml of Krebs buffer (in mM: 145 NaCl, 5 KCl, 10 HEPES/NaOH pH 7.4, 1 NaH_2_PO_4_, 2.5 CaCl_2_, 1.8 MgSO_4_, 5 glucose) through the heart. The kidneys were removed, minced, and digested in collagenase (1 mg/ml collagenase A (Worthington, Lakewood, NJ, USA), 0.6 mg/ml hyaluronidase) in Krebs buffer. The digested tubules sized between 40 and 100 μm were sorted based on GFP fluorescence by COPAS (Complex Object Parametric Analysis and Sorting, Union Biometrica, Holliston, MA, USA). Per mouse, 4,000 eGFP-positive fluorescent DCT tubules were collected, and an additional 4,000 control tubules were sorted from the same kidney sample without selection for eGFP-positive cells.

### RNA Isolation and cDNA synthesis

Total RNA was isolated using TRIzol total RNA isolation agent (Invitrogen, Bleiswijk, the Netherlands) according to the manufacturer’s protocol. Obtained RNA was precipitated in ethanol, washed, and dissolved in nuclease-free ultrapure water. RNA concentrations were measured spectrophotometrically and purity was determined: in all samples, the ratio of optical density at 260 and 280 nm wavelength was >1.8. Next, 1 μg of RNA was subjected to DNase treatment (Promega, Fitchburg, WI, USA) to prevent genomic DNA contamination. Subsequently, RNA was reverse-transcribed by Moloney Murine Leukemia Virus Reverse Transcriptase (Invitrogen, Bleiswijk, the Netherlands) according to the manufacturer’s instructions (1 h at 37 °C). Samples were then diluted 1:10 with nuclease-free ultrapure water and stored at −20 °C until further use.

### Real time quantitative PCR

Relative mRNA expression was assessed by quantitative real-time polymerase chain reaction (RT-qPCR). Primers used for RT-qPCR were designed using the Primer-BLAST tool (http://www.ncbi.nlm.nih.gov/tools/primer-blast/) and are shown in Supplementary Table 1. Two and a half μL of cDNA template and an optimal concentration (which was determined for each gene during primer validation and was of 400 nM) of forward and reverse primers were added to 6.25 μL 2 × iQ™SYBR^®^ Green supermix (Bio-Rad, Veenendaal, the Netherlands). The total volume was adjusted to 12.5 μL with diethylpyrocarbonate (DEPC)-treated deionized H_2_O. RT-qPCR (7 min at 95 °C, 40 cycles of 15 s at 95 °C and 1 min at 60 °C) was carried out using a CFX96 detection system (Bio-Rad, Veenendaal, the Netherlands). As a negative control, the cDNA template was substituted for DEPC-treated water. Additionally, to ensure that residual genomic DNA was not being amplified, control samples, in which reverse transcriptase was omitted during cDNA synthesis, were included in the plates during measurements. All samples were normalized to the expression level of the standard mouse-specific reference gene *Gapdh*^40^. Gene expression data were calculated using the Livak method (2^−ΔΔCt^) and they represent the mean fold difference from the calibrator/control group.

For primer validation, standard curves with serially diluted cDNA were generated and primer concentration was optimized to ensure the efficiencies of RT-qPCR (95–105%). The construction of SYBR Green dissociation curves after completion of 40 PCR cycles revealed the presence of single amplicons for each primer pair. Amplicon size was confirmed by electrophoresis in 1.5% (*w*/*v*) agarose gel.

### Animals

All experimental protocols and procedures involving animals were approved by the animal ethics board of Radboud University (Nijmegen, The Netherlands) and were in compliance with National and European guidelines. Heterozygous male and female (*Slc41a3*^+/–^) mice of the Slc41a3^tm1a(KOMP)Wtsi^ strain were purchased from Knock Out Mouse Project repository (KOMP, Davis, CA, USA MGI: 1918949) and crossbred to C57Bl/6N wild-type mice. The heterozygous offspring was used to generate *Slc41a3*^–/–^ mice. Littermates were housed in a temperature- and light-controlled room with standard pellet chow and deionized drinking water available *ad libitum*.

### Diet study

20 *Slc41a3*^+/+^, 20 *Slc41a3*^+/−^ and 20 *Slc41a3*^−/−^ mice aged between 8–12 weeks were randomly selected for this experiment (50% male, 50% female). The animals were housed in metabolic cages for 48hrs (24hrs adaptation, 24hrs sampling) prior to collect urine and feces. Subsequently, the mice were randomly divided to a group and fed with normal (0.19% wt/wt Mg^2+^, SSNIFF Spezialitäten GmbH, Soest, Germany) and low Mg^2+^ diets (0.02% wt/wt Mg^2+^, SSNIFF) (n = 10 per group per genotype) for 14 days. Blood samples were taken before and after the diets via submandibular facial vein puncture. The last 48hrs of the experiment the animals were housed in the metabolic cages again to collect urine and feces. Then, animals were sacrificed, blood was collected and kidney, brain and colon tissues were sampled and frozen immediately in liquid nitrogen for further analysis.

### ^25^Mg^2+^ absorption study

Intestinal absorption of Mg^2+^ was measured by analyzing serum ^25^Mg^2+^ as percentage of total Mg^2+ ^18. In short, 20 male animals aged 8–10 weeks (10 *Slc41a3*^+/+^, 10 *Slc41a3*^−/−^) were fasted (food, not water) overnight on wire-mesh raised floors to prevent coprophagia. At time-point 0, mice were administered a solution containing 44 mM ^25^Mg^2+^ (MgO, isotopic enrichment of >98%, CortectNet, Voisins-Le-Bretonneux, France), 125 mM NaCl, 17 mM Tris-HCl pH 7.5, 1.8 g/L fructose. Animals were administered a volume of 15 uL/g bodyweight via oral gavage. Subsequently, blood was taken at serial time-points via a small tail-cut and collected in Microvette serum tubes (Sarstedt, Etten-Leur, The Netherlands). After serum collection from the coagulated blood samples, sera were digested in nitric acid (65% concentrated, Sigma, Zwijndrecht, The Netherlands) for 1 hr at 70 °C followed by an overnight incubation at room temperature. Subsequently, samples were diluted in milliQ and subjected to ICP-MS analysis (X1 series, Thermo Fisher Scientific, Breda, The Netherlands).

### Histology

After the sampling, the tissues specimens were immediately fixed in 4% PFA (w/v) and embedded in paraffin or frozen in liquid nitrogen. 5μm sections were cut in embedding media (Tissue-Tek, OCT medium, Sakura, Alphen aan den Rijn, The Netherlands), mounted onto Superfrost Plus slides (Thermo Scientific, Menzel-Glaser, Braunschweig, Germany) and stored at −20 °C. Paraffin sections were deparaffinized and rehydrated and stained with a Hematoxylin and eosin (H&E) staining. Slides were put in Hematoxillin and differentiated in tapwater. Thereafter the slides were counterstained with Eosin Y, dehydrated and mounted. Pictures were made with a Zeiss (Oberkochen, Germany) microscope.

### Mg^2+^ and Ca^2+^ measurements

Serum and urine total Mg^2+^ and Ca^2+^ concentrations were determined using a colorimetric assay kit according to the manufacturer’s protocol (Roche Diagnostics, Woerden, the Netherlands). Urine volume was measured to calculate 24 hours excretion.

### Statistical analysis

Data are expressed as mean ± SEM. Statistical comparisons were analyzed by two-way ANOVA with a Tukey’s multiple comparison test. When only two experimental groups were affected by only one factor of variance, an unpaired Student’s *t*-test was used (Figs 1 and 6). P < 0.05 was considered statistically significant.

## Additional Information

**How to cite this article**: de Baaij, J. H.F. *et al*. Identification of SLC41A3 as a novel player in magnesium homeostasis. *Sci. Rep.* **6**, 28565; doi: 10.1038/srep28565 (2016).

## Supplementary Material

Supplementary Information

## Figures and Tables

**Table 1:** Metabolic parameters of *Slc41a3*^−/−^ mice.

	Normal Mg^2+^ diet			Low Mg^2+^ diet		
	*Slc41a3*^+/+^	*Slc41a3*^+/−^	*Slc41a3*^−/−^	*Slc41a3*^+/+^	*Slc41a3*^+/−^	*Slc41a3*^−/−^
Weight – day 0 (g)	18.0 ± 2.1	18.4 ± 1.6	17.5 ± 2.2	18.4 ± 2.1	18.7 ± 2.0	17.7 ± 3.2
Weight – day 14 (g)	20.5 ± 2.1	20.5 ± 1.9	20.2 ± 2.5	19.8 ± 2.6	20.3 ± 2.4	19.1 ± 3.3
Food intake (g)	3.97 ± 0.50	4.26 ± 0.23	4.43 ± 0.28	3.46 ± 0.52	3.56 ± 0.42	3.76 ± 0.56
Water intake (mL)	4.95 ± 1.23	4.98 ± 1.13	4.77 ± 0.88	4.00 ± 0.82	4.65 ± 1.12	4.03 ± 0.61
Feces weight (g)	1.86 ± 0.38	1.94 ± 0.39	2.09 ± 0.22	0.34 ± 0.12	0.38 ± 0.08	0.34 ± 0.13
Urine volume (mL)	1.25 ± 0.31	1.12 ± 0.37	1.22 ± 0.29	1.53 ± 0.66	1.61 ± 0.54	1.46 ± 0.48
Serum [Na^+^] (mmol/L)	152 ± 1	150 ± 1	153 ± 1	152 ± 0	152 ± 0	153 ± 0
Serum [K^+^] (mmol/L)	4.5 ± 0.1	4.6 ± 0.1	4.5 ± 0.1	4.2 ± 0.2	4.4 ± 0.1	4.8 ± 0.1
Urine Na^+^ (μmol/24 hrs)	185 ± 13	184 ± 20	214 ± 11	204 ± 13	169 ± 17	176 ± 26
Urine K^+^ (μmol/24 hrs)	539 ± 26	538 ± 39	562 ± 25	559 ± 39	460 ± 46	502 ± 69
