# Supplementary material for: Identification of SLC41A3 as a novel player in magnesium homeostasis
Source: Sci Rep. 2016 Jun 28;6:28565. doi: 10.1038/srep28565 (PMC4923877; doi:10.1038/srep28565)

SUPPLEMENTARY DATA

Identification of SLC41A3 as a novel player in renal magnesium homeostasis

Jeroen H.F. de Baaij[1], *Francisco J. Arjona*[1], *Michiel van den Brand*[2], *Marla Lavrijsen*[1], *Anke Lameris*[1], *René J.M. Bindels*[1], *Joost G.J. Hoenderop*[1] *

Departments of Physiology[1] and Pathology[2], Radboud Institute for Molecular Life Sciences, Radboud university medical center, Nijmegen, The Netherlands

Supplementary Table TI – Primer sequences

	Forward primer	Reverse primer
Gapdh	5’-TAACATCAAATGGGGTGAGG-3’	5’-GGTTCACACCCATCACAAAC-3’
Slc41a1	5’-CATCCCACACGCCTTCCTGC-3’	5’-CGGCTGGCCTGCACAGCCAC-3’
Slc41a2	5’-TGGCATGGTTTTGGACATAG-3’	5’-AGCGTCATTTCCAAGTTTCC-3’
Slc41a3	5’-TGAAGGGAAACCTGGAAATG-3’	5’-GGTTGCTGCTGATGATTTTG-3’
Trpm6	5’-AAAGCCATGCGAGTTATCAGC-3’	5’-CTTCACAATGAAAACCTGCCC-3’
Trpm7	5’-GGTTCCTCCTGTGGTGCCTT-3’	5’-CCCCATGTCGTCTCTGTCGT-3’
Cnnm2	5’-GGAGGATACGAACGACGTG-3’	5’-TTGATGTTCTGCCCGTACAC-3’
Cnnm4	5’-TCTGGGCCAGTATGTCTCTG-3’	5’-CACAGCCATCGAAGGTAGG-3’
Parvalbumin	5’-CGCTGAGGACATCAAGAAGG-3’	5’-AGCTTTCAGCCACCAGAGTG-3’
Egf	5’-GAGTTGCCCTGACTCTACCG-3’	5’-CCACCATTGAGGCAGTATCC-3’
Ncc	5’-CTTCGGCCACTGGCATTCTG-3’	5’-GATGGCAAGGTAGGAGATGG-3’
Cldn16	5’-GTTGCAGGGACCACATTAC-3’	5’-GAGGAGCGTTCGACGTAAAC-3’
Cldn19	5’-GGTTCCTTTCTCTGCTGCAC-3’	5’-CGGGCAACTTAACAACAGG-3’

Supplemental Figure S1 – Compensatory mechanisms for the loss of Slc41a3 function in the kidney

A-D, The mRNA expression levels of *Ncc* (A), *Egf* (B), *Cldn16* (C) and *Cldn19* (D) in kidney of *Slc41a3*$^{+/+}$ (black bars), *Slc41a3*$^{+/-}$ (striped bars) and *Slc41a3*$^{-/-}$ (white bars) mice fed with a low or normal Mg^{2+}-containing diet for 14 days were measured by RT-qPCR. Relative gene expression was analyzed using the Livak method ($2^{-\Delta\Delta Ct}$), where results are normalized against *Gapdh* expression (reference gene). Data represent means (n=10) ± SEM and are expressed as fold difference when compared to the gene expression in normal diet fed *Slc41a3*$^{+/+}$ mice. *P < 0.05 indicates a statistically significance compared to *Slc41a3*$^{+/+}$ mice fed the same diet.

Supplemental Figure S2 – Brain expression of Mg^{2+} transporters

A-B, The mRNA expression levels of *Trpm7* (A) and *Slc41a1* (B) in brain of *Slc41a3*$^{+/+}$ (black bars), *Slc41a3*$^{+/-}$ (striped bars) and *Slc41a3*$^{-/-}$ (white bars) mice fed with a low or normal Mg^{2+}-containing diet for 14 days were measured by RT-qPCR. Relative gene expression was analyzed using the Livak method ($2^{-\Delta\Delta Ct}$), where results are normalized against *Gapdh* expression (reference gene). Data represent means (n=10) ± SEM and are expressed as fold difference when compared to the gene expression in normal diet fed *Slc41a3*$^{+/+}$ mice. *P < 0.05 indicates a statistically significance compared to *Slc41a3*$^{+/+}$ mice fed the same diet.

Supplemental Figure 1

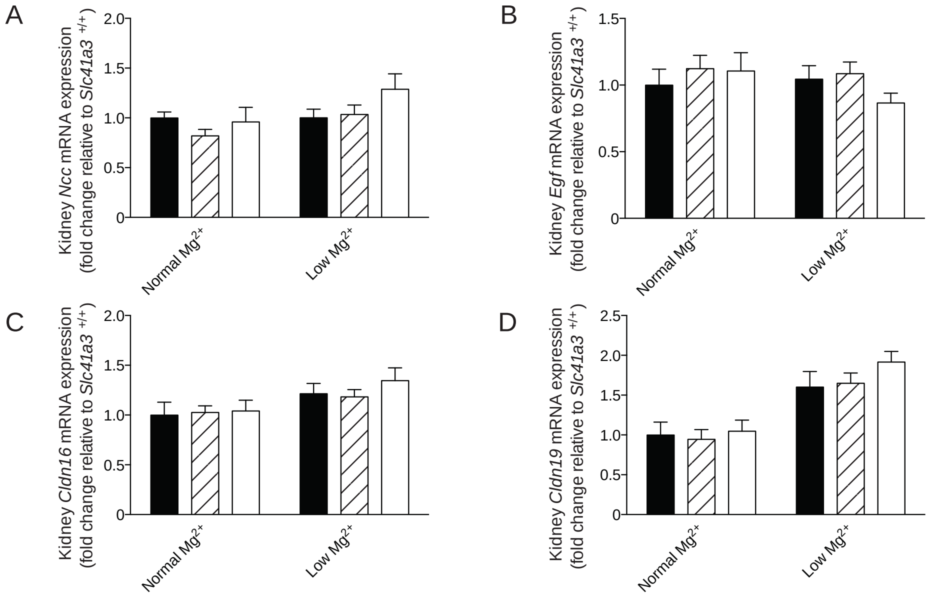

Supplemental Figure 2

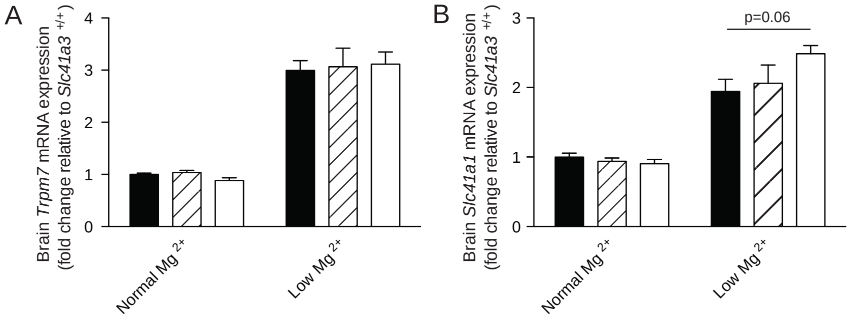

Supplement: Supplementary Information [file srep28565-s1.pdf]
